# Multi-Talker Speech Promotes Greater Knowledge-Based Spoken Mandarin Word Recognition in First and Second Language Listeners

**DOI:** 10.3389/fpsyg.2020.00214

**Published:** 2020-02-20

**Authors:** Seth Wiener, Chao-Yang Lee

**Affiliations:** ^1^Language Acquisition, Processing and Pedagogy Lab, Department of Modern Languages, Carnegie Mellon University, Pittsburgh, PA, United States; ^2^Speech Processing Lab, Communication Sciences and Disorders, Ohio University, Athens, OH, United States

**Keywords:** gating, spoken word recognition, Mandarin Chinese, talker variability, second language acquisition, distributional learning, lexical tone

## Abstract

Spoken word recognition involves a perceptual tradeoff between the reliance on the incoming acoustic signal and knowledge about likely sound categories and their co-occurrences as words. This study examined how adult second language (L2) learners navigate between acoustic-based and knowledge-based spoken word recognition when listening to highly variable, multi-talker truncated speech, and whether this perceptual tradeoff changes as L2 listeners gradually become more proficient in their L2 after multiple months of structured classroom learning. First language (L1) Mandarin Chinese listeners and L1 English-L2 Mandarin adult listeners took part in a gating experiment. The L2 listeners were tested twice – once at the start of their intermediate/advanced L2 language class and again 2 months later. L1 listeners were only tested once. Participants were asked to identify syllable-tone words that varied in syllable token frequency (high/low according to a spoken word corpus) and syllable-conditioned tonal probability (most probable/least probable in speech given the syllable). The stimuli were recorded by 16 different talkers and presented at eight gates ranging from onset-only (gate 1) through onset +40 ms increments (gates 2 through 7) to the full word (gate 8). Mixed-effects regression modeling was used to compare performance to our previous study which used single-talker stimuli (Wiener et al., 2019). The results indicated that multi-talker speech caused both L1 and L2 listeners to rely greater on knowledge-based processing of tone. L1 listeners were able to draw on distributional knowledge of syllable-tone probabilities in early gates and switch to predominantly acoustic-based processing when more of the signal was available. In contrast, L2 listeners, with their limited experience with talker range normalization, were less able to effectively transition from probability-based to acoustic-based processing. Moreover, for the L2 listeners, the reliance on such distributional information for spoken word recognition appeared to be conditioned by the nature of the acoustic signal. Single-talker speech did not result in the same pattern of probability-based tone processing, suggesting that knowledge-based processing of L2 speech may only occur under certain acoustic conditions, such as multi-talker speech.

## Introduction

Bilingual lexical processing is typically described as plastic or flexible. As an example, bilingual listeners activate lexical candidates in both their first (L1) and second (L2) language; yet despite the increased competition, bilinguals demonstrate remarkable flexibility in their ability to recognize spoken words (Spivey and Marian, 1999; Weber and Cutler, 2004, 2006; Chang, 2016; see Chen and Marian, 2016; Fricke et al., 2019 for reviews). Whereas recent research has primarily explored this flexibility in terms of the cognitive resources involved in spoken word recognition, such as inhibitory control and working memory (e.g., Blumenfeld and Marian, 2011, 2013; Kroll and Bialystok, 2013; Linck et al., 2014), here we explore this flexibility in terms of listeners’ ability to navigate between the acoustic signal and learned distributional information about the likelihood of speech sounds. Specifically, we examine how acoustic-based and knowledge-based L2 spoken word recognition occurs under adverse listening conditions involving multi-talker, high variability speech.

How listeners accomplish perceptual constancy in the face of acoustic variability is one of the primary puzzles in speech perception and spoken word recognition (e.g., Mullennix et al., 1989; Johnson, 2005; Mattys et al., 2012; Guediche et al., 2013). An understanding of how acoustic variability affects L1 and L2 speech perception further clarifies theoretical accounts of how linguistic knowledge contributes to speech perception (Lecumberri et al., 2010). Likewise, how and to what extent adult L2 learners become “native-like” in their perceptual abilities over time remains crucial to our theoretical understanding of second language acquisition and bilingualism (Flege, 1995; Escudero and Boersma, 2004; Best and Tyler, 2007). To this end, in addition to examining acoustic-based and knowledge-based processing of multi-talker speech, we examine how these modes of processing change as listeners become more proficient in their L2 abilities. We use Mandarin Chinese as the target language of this study and test adult L1 and L2 Mandarin listeners, the latter group we consider to be unbalanced or L1 dominant bilinguals undergoing structured L2 classroom learning at the time of testing.

### Acoustic-Based Mandarin Spoken Word Recognition

To recognize a spoken Mandarin word, listeners must perceive segmental (i.e., consonants and vowels) and suprasegmental (i.e., tones) information and map this combination to a stored lexical representation. The primary acoustic correlate of tone is fundamental frequency (F0) though secondary duration and amplitude cues also play a role in tone perception (Howie, 1976; Blicher et al., 1990; Whalen and Xu, 1992; Moore and Jongman, 1997). Tones can constrain online word recognition in a manner analogous to segments (Malins and Joanisse, 2010, 2012). For example, the syllable *da* with a high-level F0 (Tone 1) means “to put up/hang”; *da* with a mid-rising F0 (Tone 2) means “to attain/reach”; *da* with a low-dipping F0 (Tone 3) means “to hit/beat”; and *da* with a high-falling F0 (Tone 4) means “big/large.” Importantly, the phonetic correlates of tone are highly variable and affected by numerous factors including coarticulation, contextual variation, local and global prosodic structures, and, the focus of the present study: talker variability (Leather, 1983; Speer et al., 1989; Fox and Qi, 1990; Xu, 1994, 1997; Wong and Diehl, 2003; Lee, 2009; Lee et al., 2010a).

Ample evidence shows that linguistic experience affects the way acoustic cues are processed. For example, L1 and L2 listeners differ in the way they use F0 height and F0 direction cues (Gandour, 1983; Gottfried and Suiter, 1997). L1 English-L2 Mandarin listeners, for example, tend to initially weight F0 height cues greater than F0 direction cues; this results in perceptual confusion between tones with high F0 onsets – Tone 1 and 4 – and tones with low F0 onset – Tone 2 and 3 (Wang et al., 1999; Chandrasekaran et al., 2010; Hao, 2012, 2018; Wiener, 2017). Though, it is worth noting even L1 Mandarin listeners who heavily weight F0 direction still demonstrate some confusion between Tone 2 and 3 given their similar contours (Shen and Lin, 1991; Shen et al., 2013).

Crucial to the present study, L1 and L2 listeners also differ in the way they process acoustic variability. Given L2 listeners’ imperfect knowledge of the target language, it is perhaps not surprising that L2 listeners are often disproportionately affected by acoustic variability. In terms of lexical tone perception, fragmented acoustic input, such as silent-center syllables (Strange et al., 1983), affects L1 and L2 tone perception differently. Whereas L1 listeners are able to integrate tonal information from the initial and final portions of a silent-center syllable to reconstruct the intended tones, L2 listeners do not take advantage of the dynamic tonal information in the remaining fragments of the syllable (Gottfried and Suiter, 1997; Lee et al., 2008, 2010b). Similarly, when presented with tones excised from the original context and cross-spliced onto a new context, L1 listeners but not L2 listeners, show sensitivity to such contextual tonal variations (Gottfried and Suiter, 1997; Lee et al., 2008, 2010b). Finally, mixing noise with tonal stimuli compromises both L1 and L2 tone perception, but it affects L2 tone perception disproportionately (Lee et al., 2009, 2010c, but see Lee et al., 2013 for an alternative analysis).

In contrast with these aforementioned sources of acoustic variability, talker variability appears to affect L1 and L2 tone perception similarly. Studies using multi-talker stimuli have shown that tone perception in both L1 and L2 listeners is adversely affected by talker variability to roughly similar extents (Lee et al., 2009, 2010b, 2013). Although unexpected, the absence of a disproportionate effect of talker variability on L2 tone perception is actually consistent with what has been found in the literature on segmental recognition in spoken words (Bradlow and Pisoni, 1999; Takayanagi et al., 2002; Lee et al., 2012). It is also of interest to note that high-variability training involving stimuli from multiple talkers usually results in more effective tone learning by L2 listeners than stimuli from a single-talker (Wang et al., 1999; Perrachione et al., 2011; Sadakata and McQueen, 2014; Zhang K. et al., 2018, but see also Dong et al., 2019).

Although talker variability appears to affect L1 and L2 tone perception similarly, there is evidence that L1 and L2 listeners process talker variability differently when the acoustic cues typically considered necessary for talker normalization are absent – a situation presented in the current study. Since tone perception depends primarily on the processing of F0, and F0 range differs across talkers, tone perception usually involves estimating a talker’s F0 range (Leather, 1983; Moore and Jongman, 1997; Wong and Diehl, 2003). Several studies show that L1 listeners are able to identify tones without typical normalization cues such as context, familiarity with talkers, and F0 contour. Lee (2009), for example, presented Mandarin /sa/ syllables with all four tones produced by 16 female and 16 male speakers. Each stimulus was presented in isolation and only once in a random order. In addition, only the fricative and the first six glottal periods were present in the stimuli, effectively neutralizing the F0 contour contrasts among the tones. Despite the absence of normalization cues, L1 Mandarin listeners were able to identify the tones with above-chance accuracy. Similarly, Lee et al. (2011) presented two Taiwanese syllables /hue/ and /di/ with two level tones (high and mid) produced by 15 male and 15 female speakers. The task was to identify whether a stimulus was intended as a high or mid tone. Despite the speaker sex and range differences, L1 Taiwanese listeners were able to identify all four categories – female/high-tone, female/low-tone, male/high-tone, and male/low-tone – with above-chance accuracy.

In contrast, a different pattern of talker normalization emerged for L2 listeners when Lee et al. (2014) presented the same Taiwanese stimuli to L2 English listeners. Unlike the L1 listeners in Lee et al. (2011), the L2 listeners could only identify stimuli at the extremes of the talkers’ F0 range (i.e., female/high-tone & male/low-tone) but not those in the middle of the range (i.e., female/low-tone & male/high-tone). Taken together, these findings suggest that whereas both L1 and L2 listeners are able to use syllable-internal information for talker normalization in the absence of contextual/familiarity/contour cues, L1 listeners appear to be more effective at calibrating acoustic input according to sex-specific, internally stored pitch templates (Lee, 2017).

In sum, research examining the acoustic-based processing of lexical tones in adverse conditions shows that not all sources of acoustic variability are equally disruptive to L1 and L2 tone perception. It appears that L2 tone perception is compromised disproportionately only when syllable-internal, canonical F0 information is removed or altered (Lee, 2017; Lee and Wiener, unpublished). This proposal is consistent with the observation that L2 listeners rely primarily on syllable-internal, canonical F0 information for tone perception, whereas L1 listeners are more capable of using knowledge of tonal coarticulation and contextual tonal variation to compensate for missing F0 information. When syllable-internal, canonical F0 information is reduced (as in fragmented acoustic input), altered (as in syllables excised from the original tonal context), or compromised (as in noise), L2 tone perception tends to be disrupted disproportionately. In contrast, talker variability, which presents variability in the form of different F0 ranges across talkers, does not affect L2 tone perception disproportionately most likely because it does not remove or alter syllable-internal, canonical F0 information (Lee et al., 2009, 2010b, 2013). L1 and L2 listeners, however, differ in their ability to identify tones in the absence of acoustic cues typically considered necessary for talker normalization.

### Knowledge-Based Mandarin Spoken Word Recognition

Mandarin provides clear distributions of speech sounds, which are traced and exploited by native and non-native listeners (e.g., Fox and Unkefer, 1985; Wang, 1998; Wiener and Ito, 2015; Wiener and Turnbull, 2016; Wiener et al., 2019). For the majority of speech tokens, there exists a relatively straightforward mapping from the syllable, to the morpheme, to the word, to the written character without the need for intervening derivational morphology (Zhou and Marslen-Wilson, 1994, 1995; Packard, 1999, 2000; Myers, 2006, 2010; see also Tao, 2015 for corpus evidence). As an example, the word for “big” is one syllable, morpheme, and character: *da4* 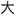.

At the syllable level, there are approximately 400 unique (C)V(C) syllable types (where C and V refer to consonant and vowel, respectively), each of which differs in token frequency (De Francis, 1986; Duanmu, 2007, 2009). For example, for every *fo* syllable token in the 33.5 million spoken word corpus SUBTLEX–CH (Cai and Brysbaert, 2010) there are nearly 300 *wo* syllable tokens. At the morpheme level, each syllable can co-occur with one of the four phonemic tones. Yet, not all syllables co-occur with all four tones. The syllable *fo*, for instance, only occurs with Tone 2. When *fo* is produced with the other three tones, the combination results in a non-word (analogous to “blick” in English). At the word/character level, each syllable-tone combination can represent one or more semantically and orthographically distinct forms. Within SUBTLEX-CH, *fo2* only appears as 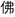 meaning “Buddha” whereas *wo* produced with Tone 4 appears as seven unique words, including 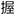 “to hold” and 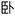 “to crouch” among others. For the purposes of the present study, we use “word” hereafter to refer to a particular monosyllabic Mandarin (C)V(C) syllable-tone combination and “syllable” to refer to a particular (C)V(C) segmental string irrespective of its tone.

Previous research has explored how such distributional information is relied upon during spoken word recognition, particularly when the acoustic signal is limited or unreliable. In a gating study – the paradigm that we use in the present study – Wiener and Ito (2016) examined how L1 Mandarin talkers navigate between truncated acoustic information and their expectation of likely Mandarin speech sounds given their distributional knowledge of the language. Participants heard a word’s onset only (gate 1), followed by 40 millisecond (ms) increments (gates 2 through 7) until the full word was heard (gate 8). Importantly, the stimuli crossed syllable token frequencies (high F+/low F−) with syllable-conditioned tonal probabilities (most probable P+/least probable P−) resulting in four conditions. For example, within SUBTLEX-CH, the high token frequency syllable *bao* is most likely to occur with tone 4 (roughly 50% chance) and least likely to occur with tone 2 (roughly 1% chance).

The authors found that from gate 2 (onset + 40 ms) until gate 5 (onset + 160 ms), L1 Mandarin listeners identified syllable-tone words consisting of high token frequency (F+) syllables more accurately than syllable-tone words consisting of low token frequency (F−) syllables. No difference was found in gates 6, 7, or 8. The frequency effect thus surfaced only when the acoustic input was not sufficiently reliable, i.e., under challenging listening conditions (e.g., Grosjean, 1980; Tyler, 1984).

An analysis of participants’ correct-syllable-incorrect-tone responses further revealed two types of tonal errors: acoustic-based and probability-based. Acoustic-based errors reflected listeners’ use of limited cues such as F0 height to report a perceptually similar tone, e.g., upon hearing *bao2*, reporting *bao3* due to the two tones’ similar low F0 onset. Probability-based errors, on the other hand, reflected listeners’ use of syllable-conditioned tonal probabilities, e.g., upon hearing *bao2* reporting *bao4* due to its far more probable occurrence in speech, even if it was acoustically incongruent with the limited acoustic cues. In the first three gates (onset + up to 80 ms of the vowel) participants overall made more probability-based errors than acoustic-based errors, suggesting listeners relied on syllable-conditioned tonal probabilities when the acoustic signal was not sufficient to allow for tonal decisions. However, this difference was statistically significant only for low token frequency (F−) syllables, indicating the tendency to use tonal probabilities is particularly evident when dealing with less commonly encountered words. The authors argued that the two-way token frequency and tonal probability interaction was likely driven by homophone density: tone is more informative given fewer lexical candidates within sparse neighborhoods (e.g., Li and Yip, 1998; Yip, 2000; Chen et al., 2009; Wiener and Ito, 2015).

To explore whether L2 learners also utilize distributional knowledge of tonal probabilities in Mandarin word recognition and how that knowledge develops over time, Wiener et al. (2019) extended Wiener and Ito (2016) by testing L1 English-L2 Mandarin adult classroom learners at two time points roughly 3 months apart (along with a control group of L1 listeners tested once). New test items that were more appropriate for the L2 learners were used. The L1 listeners performed identically to those tested in Wiener and Ito (2016). That is, in addition to higher accuracy for high-frequency words, L1 listeners showed more probability-based errors in early gates. Like the L1 listeners, the L2 listeners drew on syllable token frequency information in a native-like manner in the first five gates. The L2 listeners also showed a trend of improvement 3 months later in both word and tone identification; though the difference between the two tests was not statistically significant. The correct-syllable-incorrect-tone error analysis revealed that the L2 listeners did not use probability information at test 1. At test 2 3 months later, L2 listeners showed a marginal trend to respond with more probability-based errors.

In sum, evidence from the two gating studies (Wiener and Ito, 2016; Wiener et al., 2019) indicates that when the acoustic signal is limited or unreliable, both L1 and L2 Mandarin listeners rely on learned syllable token frequency information to improve their recognition of spoken words. This syllable token frequency effect in which (F+) targets are identified more accurately than (F−) targets has been consistently observed in the first five gates (onset plus up to 160 ms of the vowel) across two populations of L1 listeners and a population of L2 listeners at two different points in their L2 learning. L1 listeners also draw on tonal probability information to predict more probable tones (P+) in early gates, particularly for less frequent words within sparse neighborhoods. L2 learners with limited lexicons show a trend in which greater experience with the language prompts greater reliance on learned tonal probabilities. However, a robust effect of probability-based processing has yet to be observed in L2 listeners.

### The Present Study

The absence of robust evidence for L2 listeners’ use of tonal probability information in Wiener et al. (2019) suggests two possibilities. First, the L2 listeners tested simply did not possess sufficient tonal distributional knowledge to affect their processing of truncated tones; their knowledge of syllable-tone combinations was neither fully established at test 1 nor at test 2. Alternatively, the specific adverse listening condition tested (fragmented acoustic input) was not sufficiently effective to reveal a robust effect of probability-based processing despite the precise control of the amount of the acoustic signal presented. Because the stimuli used in Wiener et al. (2019) were produced by a single talker, once participants identified the talker’s F0 range, acoustic-based processing became relatively straightforward without the need for talker normalization.

In the current study we include talker variability as a second source of acoustic variability to further explore how acoustic-based and knowledge-based processing interact with each other in Mandarin word recognition by L1 and L2 listeners. As noted earlier, not all sources of acoustic variability affect L1 and L2 tone perception equally. Talker variability is unique in that it does not seem to compromise L2 tone perception disproportionately. We therefore examine whether a more variable acoustic signal – and thus less reliable F0 cues due to talker differences – causes L1 and L2 listeners to increase their reliance on their previous learned knowledge of likely speech sounds as a way to overcome poor acoustic-based processing. We predict that talker variability will compromise overall identification accuracy for L1 and L2 listeners (e.g., Mullennix et al., 1989; Johnson, 2005; Lee, 2017). We also predict that the syllable token frequency effect will resemble that observed in both Wiener and Ito (2016) and Wiener et al. (2019).

More important, and more interesting, is how the presence of talker variability affects L1 and L2 listeners’ use of tonal probability information, as measured by the proportion of acoustic- vs. probabilistic-based errors in Wiener et al. (2019). For L1 listeners, talker variability in the stimuli is expected to trigger more probability-based processing at early gates given the less reliable acoustic signal. L1 listeners should also gravitate toward predominately acoustic-based processing at late gates when more tonal information becomes available and L1 listeners can rely on their superior experience with talker normalization to decipher multi-talker tones.

For L2 listeners, multi-talker speech could affect L2 listeners’ performance in two ways. On the one hand, because talker variability affects L1 and L2 similarly (Lee et al., 2009, 2010b, 2013), because L2 listeners have ample experience with talker normalization in their native language, and because L2 listeners show less robust evidence of probability-based processing (Wiener et al., 2019), the presence of talker variability may not change the pattern of L2 performance from that observed in Wiener et al. (2019), that is, L2 listeners may predominately rely on acoustic-based processing in early and late gates and only show more probability-based processing at test 2. On the other hand, because L2 listeners possess less experience with multi-talker Mandarin speech and hence are less capable of deciphering F0 information given the range difference in multiple talkers, multi-talker speech on top of fragmented acoustic input may prompt L2 listeners to rely more on their knowledge of tonal distributions throughout the gates, however, limited this knowledge may be. This behavioral pattern may be particularly evident at test 2 when L2 knowledge is more robust. Such results would imply that L2 listeners possess some form of tonal distributions knowledge but primarily utilize the knowledge under certain acoustically challenging conditions.

## Materials and Methods

### Participants

The L1 Mandarin group consisted of 15 listeners from mainland China (10 female; 5 male; mean age = 23.4). All L1 Mandarin participants spoke English as an L2, had completed up to high school in China, and self-reported only speaking Mandarin (i.e., no other Chinese dialect). The L2 Mandarin group consisted of 15 L1 American English listeners from the United States (8 female; 7 male; mean age = 20.3) who were enrolled in a university intermediate or advanced L2 Mandarin class at the time of testing and had been studying Mandarin for an average of 3.6 years. All 30 participants were undergraduate or graduate students at a midwestern university in the United States with self-reported normal speech, hearing, and language. All were screened for normal hearing, defined as pure-tone, air-conducted thresholds of ≤20 dB HL at octave frequencies from 1000 Hz to 4000 Hz. All participants were paid or given class credit for their participation.

The 30 participants (15 L1; 15 L2) tested in Wiener et al. (2019) served as the single-talker groups used for comparison in our statistical analyses. These participants were recruited from the same respective university populations of L1 and L2 Mandarin students and followed the same testing procedure used in the present study. Only the number of talkers used to record the stimuli differed between the two groups.

### Stimuli

The 48 items designed for Wiener et al. (2019) were used for the present study (see Supplementary Material for stimuli). The stimuli were thus specifically designed to reflect words the L2 learners were familiar with given their Chinese textbooks. The stimuli consisted of 24 unique consonant-vowel syllables with an optional nasal. Each syllable varied in syllable token frequency according to calculations based on SUBTLEX–CH (Cai and Brysbaert, 2010; 12 high frequency, F+; 12 low frequency, F−). For each syllable, the most probable (occurring in over 50% of the words with that syllable, P+) and least probable (occurring in less than 25% of the words with that syllable, P–) tones were identified according to SUBTLEX-CH calculations and verified with the L2 learners’ textbook vocabulary (see Wiener and Ito, 2015 for discussion on estimating probabilities). This design resulted in 12 items in each of the four conditions: F+P+, F+P−, F−P+, F−P−. Across the conditions, Tone 1 was the target 12 times, Tone 2 was the target 11 times, Tone 3 was the target 12 times, and Tone 4 was the target 13 times. Given the constraints on choosing stimuli that the L2 learners were familiar with, there was an unavoidable correlation between syllable token frequency and homophone density (*r* = 0.61, *p* < 0.01). Additionally, the ratio of obstruents to sonorants in the onset position was not controlled. For this reason, we do not carry out a gate-by-gate tone identification analysis (cf. Wiener and Ito, 2016).

Each item was recorded by 16 different talkers (8 male; 8 female) for a total of 768 unique utterances. Talkers were from different regions of China but self-reported only speaking Mandarin (see Supplementary Material for talker information). Recordings were made in a sound-attenuated booth at 44,100 Hz. To ensure that each gate captured the same amount of acoustic information across talkers, each talker’s mean duration for each tone type was first calculated. Next, an overall mean duration was calculated for each tone type and all recordings were normalized to the following durations: 550 ms (Tone 1), 580 ms (Tone 2), 690 ms (Tone 3), 400 ms (Tone 4). These word durations preserved the intrinsic differences observed in natural speech by ensuring Tone 4 carried the shortest duration and Tone 3 carried the longest (e.g., Ho, 1976; Howie, 1976; Blicher et al., 1990; Moore and Jongman, 1997). As a result of the normalization process, some utterances increased in duration while other utterances decreased in duration. Utterances which increased or decreased in duration beyond 2.5 standard deviations from a talker’s mean were removed (approximately 8%). Amplitude across tone types was normalized following the same averaging procedure by talker and tone type to ensure that Tone 4 had the highest overall amplitude and Tone 3 had the lowest (e.g., Whalen and Xu, 1992): 68.1 dB SPL (Tone 1), 65.5 dB SPL (Tone 2), 64.3 dB SPL (Tone 3), 72.1 dB SPL (Tone 4). We note that our amplitude normalization was applied to the overall amplitude and thus did not affect the amplitude envelope within each syllable, which has been shown to be secondary cues for tone recognition (Whalen and Xu, 1992).

Each of the 48 words was fragmented into eight gates using Praat (Boersma and Weenink, 2019) with the first gate capturing the onset and the first regular periodic cycle of the vowel (at zero crossing) but no F0 information. The mean gate 1 duration was 12 ms (minimum = 6 ms; maximum = 17 ms). Mean gate 1 durations did not differ across manner of articulations (*p* = 0.83). Gates 2 through 7 involved the onset plus 40 ms increments. Gate 8 contained the full syllable-tone target. At each gate, items were pseudo-randomly selected such that participants heard all 16 talkers at each gate with each talker producing three different syllables. The order of items and talkers were randomized within and across each gate. Across the full experiment, no talker produced the same syllable-tone target twice.

### Procedure

Testing took place in a quiet lab using Superlab 5 (Haxby et al., 1993). Participants heard a stimulus over headphones and were asked to type the perceived syllable-tone word using *Pinyin* romanization. Participants were told to guess if unsure. Chinese characters were not used because keyboard character input is frequency ordered and does not require overt tone decisions. Stimuli were presented in a duration block manner across eight blocks/gates. This approach allowed for an estimated isolation point of segments (phonemes/syllables), suprasegmentals (tones), and their combination as words (see Cotton and Grosjean, 1984 for gating overview). The experiment lasted roughly 40–60 min and included participants filling out the Language Experience and Proficiency Questionnaire (LEAP-Q; Marian et al., 2007) and completing the Tonometric adaptive pitch test (Mandell, 2019). The Tonometric test required participants to listen to two successive pure tones and indicate via keyboard press whether the second tone was higher or lower in pitch relative to the first tone. This test provided an estimated pitch threshold for participants and allowed for screening of congenital amusia, which has been shown to affect normalization of lexical tones (e.g., Zhang C. et al., 2018). All participants were able to reliably discriminate between two pure tones at 22 Hz or lower.

L1 Mandarin participants were only tested once. L1 English-L2 Mandarin participants were tested twice: once at the start of their intermediate/advanced class and once roughly 2 months later. At the conclusion of the first test, L2 participants were not made aware they would be asked to return to the lab for a second test. Between the two tests, participants spent three and a half hours per week in class for approximately 30 classroom hours. On average, participants reported spending 4 outside hours per week self-studying and completing assignments. Between the two tests, the L2 learners were exposed to all the syllables tested. Although we cannot verify whether the L2 participants heard all 48 of the test items, we assume the majority of the items were heard in class, especially those with high probability (P+) tones.

## Results

### Correct Syllable-Tone Word Recognition

**FIGURE 1:**
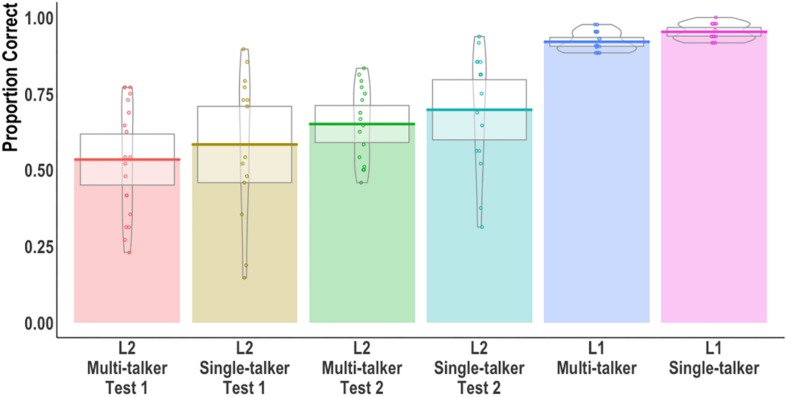
Proportion of correct syllable-tone words at gate 8 (full acoustic signal). Individual participant means (points), group means (solid line), 95% confidence intervals (white box), and group density (violin) are shown.

We first examined word recognition accuracy at gate 8, i.e., the full acoustic signal. Figure 1 shows individual participant means (points), group means (solid line), 95% confidence intervals (white box), and group density (violin). On average, each multi-talker group performed less accurately than the corresponding single-talker group. The L2 group showed similar improvements across tests seemingly independent of talker variability. To test whether group accuracy differences were observed, a mixed effects logistic regression model was built using the *lme4* package (version 1.1.21; Bates et al., 2015) in R version 3.61. The inclusion of fixed effects (and their interactions) for all models in this paper were based on the χ^2^–test of the change in deviance between the model with and without the predictor of interest (see Supplementary Material for all model comparisons; maximal random effects were included so long as the model converged). A six-level Group factor was created corresponding to the L1/L2 groups, tests, and talker condition (see Figure 1’s *x*-axis). The L1 Mandarin single-talker condition served as the reference level. Pairwise comparisons between groups were obtained using estimated marginal means (with Tukey adjusted *p*-values) obtained from the *emmeans* package (Lenth and Lenth, 2018). Table 1 reports the model output, R code, and pairwise comparisons of interest.

**TABLE 1:** Mixed effect logistic regression model on correct syllable-tone at Gate 8.

	**Estimate**	**Std. error**	***Z***	***p***
(Intercept)	3.54	0.34	10.23	<0.001
L1 Multi-talker	−0.96	0.43	−2.18	0.029
L2 Multi-talker Test 1	−3.26	0.39	−8.26	<0.001
L2 Multi-talker Test 2	−2.99	0.40	−7.50	<0.001
L2 Single-talker Test 1	−3.26	0.42	−7.67	<0.001
L2 Single-talker Test 2	−2.54	0.43	−5.96	<0.001
**Pairwise comparisons**				
L1 Multi-talker – L2 Multi-talker Test 1	2.30	0.34	6.74	<0.001
L1 Multi-talker – L2 Multi-talker Test 2	2.03	0.34	5.87	<0.001
L1 Multi-talker – L2 Single-talker Test 1	2.29	0.37	6.11	<0.001
L1 Multi-talker – L2 Single-talker Test 2	1.58	0.37	4.18	<0.001
L2 Multi-talker Test 1 – L2 Multi-talker Test 2	−0.26	0.11	−2.27	0.207
L2 Multi-talker Test 1 – L2 Single-talker Test 1	−0.01	0.36	−0.01	0.999
L2 Multi-talker Test 2 – L2 Single-talker Test 2	−0.45	0.37	−1.25	0.809
L2 Single-talker Test 1 – L2 Single-talker Test 2	−0.72	0.39	−1.83	0.443

*Glmer[corect ∼ group + (1|subject) + (talker|token), family = “binomial”]. Reference level = L1 Single-talker.*

A main effect of Group was found: the two L1 groups were more accurate than all L2 groups irrespective of test or talker condition. The L1 single-talker group was additionally more accurate than the L1 multi-talker group. Inspection of the L1 errors revealed that the multi-talker group made more syllable [χ^2^(1) = 8.03, *p* < 0.01] and tone [χ^2^(1) = 9.34, *p* < 0.01] errors compared to the single-talker group. The single-talker and multi-talker L2 groups did not differ from one another at test 1 or at test 2. Neither L2 group showed a statistically significant accuracy increase between tests.

**FIGURE 2:**
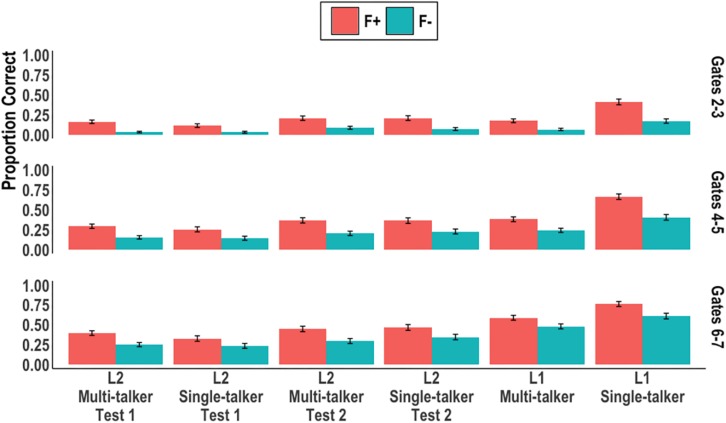
Proportion of correct syllable-tone words at gates 2–3, 4–5, and 6–7 by syllable token frequency and group. Error bars represent 95% confidence intervals.

We next examined word recognition accuracy across the early gates. Figure 2 summaries the results from gates 2 through 7 by plotting mean correct syllable-tone accuracy by syllable token frequency, gates, and groups. Following Wiener et al.’s (2019) approach to increase statistical power, three models were built with each model containing data from two consecutive gates: gates 2–3, gates 4–5, gates 6–7. Each model contained the aforementioned six-level factor, Group, and the contrast coded predictor Frequency (+1, −1) which represented syllable token frequency. Table 2 reports the model output, R code, and pairwise comparisons of interest for each model.

**TABLE 2:** Mixed effect logistic regression model on correct syllable-tone, Gates 2-3, 4-5, 6-7.

**Gates 2-3**	**Estimate**	**Std.**	***Z***	***P***
		**error**		
(Intercept)	−1.42	0.28	−4.99	<0.001
L1 Multi-talker	−1.48	0.29	−5.03	<0.001
L2 Multi-talker Test 1	−1.65	0.29	−5.70	<0.001
L2 Multi-talker Test 2	−1.12	0.29	−3.82	<0.001
L2 Single-talker Test 1	−2.06	0.32	−6.38	<0.001
L2 Single-talker Test 2	−1.18	0.31	−3.76	<0.001
Frequency	0.78	0.18	4.23	<0.001
**Pairwise comparisons**				
L1 Multi-talker – L2 Multi-talker Test 1	0.18	0.27	0.66	0.986
L1 Multi-talker – L2 Multi-talker Test 2	−0.36	0.27	−1.31	0.781
L1 Multi-talker – L2 Single-talker Test 1	0.59	0.30	1.91	0.395
L1 Multi-talker – L2 Single-talker Test 2	−0.29	0.29	−0.99	0.922
L2 Multi-talker Test 1 – L2 Multi-talker Test 2	−0.53	0.11	−2.53	0.100
L2 Multi-talker Test 1 – L2 Single-talker Test 1	0.40	0.30	1.33	0.763
L2 Multi-talker Test 2 – L2 Single-talker Test 2	0.06	0.29	0.21	0.999
L2 Single-talker Test 1 – L2 Single-talker Test 2	−0.88	0.32	−2.68	0.078
**Gates 4–5**				
(Intercept)	0.15	0.27	0.54	585
L1 Multi-talker	−1.28	0.29	−4.36	<0.001
L2 Multi-talker Test 1	−1.91	0.29	−6.53	<0.001
L2 Multi-talker Test 2	−1.61	0.29	−5.49	<0.001
L2 Single-talker Test 1	−2.11	0.32	−6.59	<0.001
L2 Single-talker Test 2	−1.41	0.32	−4.46	<0.001
Frequency	0.55	0.16	3.34	<0.001
**Pairwise comparisons**				
L1 Multi-talker – L2 Multi-talker Test 1	0.63	0.267	2.36	0.170
L1 Multi-talker – L2 Multi-talker Test 2	0.34	0.26	1.25	0.810
L1 Multi-talker – L2 Single-talker Test 1	0.83	0.30	2.79	0.058
L1 Multi-talker – L2 Single-talker Test 2	0.14	0.29	0.46	0.997
L2 Multi-talker Test 1 – L2 Multi-talker Test 2	−0.29	0.09	−2.24	0.190
L2 Multi-talker Test 1 – L2 Single-talker Test 1	0.20	0.29	0.68	0.983
L2 Multi-talker Test 2 – L2 Single-talker Test 2	−0.20	0.29	−0.67	0.984
L2 Single-talker Test 1 – L2 Single-talker Test 2	−0.69	0.32	−2.16	0.255
**Gates 6–7**				
(Intercept)	1.02	0.26	3.97	<0.001
L1 Multi-talker	−0.84	0.27	−3.05	0.002
L2 Multi-talker Test 1	−2.05	0.27	−7.50	<0.001‘
L2 Multi-talker Test 2	−1.89	0.28	−6.86	<0.001
L2 Single-talker Test 1	−2.32	0.30	−7.76	<0.001
L2 Single-talker Test 2	−1.56	0.30	−5.23	<0.001
Frequency	0.37	0.15	2.45	0.014
**Pairwise comparisons**				
L1 Multi-talker – L2 Multi-talker Test 1	1.21	0.25	4.90	<0.001
L1 Multi-talker – L2 Multi-talker Test 2	1.05	0.25	4.22	<0.001
L1 Multi-talker – L2 Single-talker Test 1	1.49	0.27	5.38	<0.001
L1 Multi-talker – L2 Single-talker Test 2	0.72	0.27	3.62	0.012
L2 Multi-talker Test 1 – L2 Multi-talker Test 2	−0.16	0.084	−1.88	0.416
L2 Multi-talker Test 1 – L2 Single-talker Test 1	0.27	0.27	0.99	0.922
L2 Multi-talker Test 2 – L2 Single-talker Test 2	−0.34	0.27	−1.23	0.819
L2 Single-talker Test 1 – L2 Single-talker Test 2	−0.77	0.30	−2.58	0.103

*Glmer[corect ∼ group + frequency + (frequency| subject) + (talker| token), family = “binomial”]. Reference level = L1 Single-talker.*

In the first two models (gates 2-3 and gates 4-5) the same pattern was observed: main effects of Group and Frequency were found. The L1 single-talker group was more accurate than all other groups; all four L2 groups and the L1 multi-talker group were statistically similar. The Frequency effect was consistent across all groups in each model (*p*s < 0.01); syllable-tone words consisting of high frequency syllables (F+) were identified more accurately than those consisting of low frequency syllables (F−), thus replicating the frequency effect found in Wiener and Ito (2016) and Wiener et al. (2019). *Post hoc* analyses into the L1 groups revealed the difference in performance was driven by a two-way interaction between syllable token frequency and talker variability (gates 2-3: β = −0.11, SE = 0.05, *Z* = −2.06, *p* = 0.03; gates 4-5: β = −0.59, SE = 0.17, *Z* = −3.45, *p* < 0.001): the single-talker group was more than twice as accurate at recognizing (F+) words compared to the multi-talker group. The two L1 groups, however, did not differ in their recognition of (F−) words (*p*s > 0.05).

In the last model (gates 6-7), main effects of Group and Frequency were again found. The L1 single-talker group remained more accurate than the L1 multi-talker group; the L1 single-talker and multi-talker groups were both more accurate than all the L2 groups; the L2 groups did not differ from one another in accuracy. The Frequency effect was found to be an aggregate effect across all listeners; no group revealed a significant difference between (F+) and (F−) targets at an alpha-level of.05.

Thus, multi-talker speech affected L1 and L2 Mandarin listeners differently in terms of their correct recognition of syllable-tone words. For L1 listeners, multi-talker speech led to less accurate word recognition across all tested gates when compared to single-talker speech. In the early gates, this difference was primarily driven by disproportionately reduced (F+) target accuracy in the multi-talker condition. For L2 listeners, multi-talker speech did not affect correct responses at any of the gates tested when compared to single-talker speech nor did it interact with word syllable frequency or test.

### Correct Syllable-Incorrect Tone Errors

We next examined errors in which listeners correctly identified the syllable but incorrectly identified the tone and therefore made either an acoustic-based or probability-based error. Acoustic-based errors stemmed from reporting an acoustically similar tone (such as *bao3* instead of *bao2*) given the two tones’ similar starting F0 heights. Probability-based errors stemmed from reporting the most probable tone given the perceived syllable (such as *bao4* instead of *bao2*). Responses in which it was unclear whether the error was acoustic-based or probability-based and responses in which the error was neither (such as reporting *bao1* instead of *bao2*) were removed (20%). This left 2,929 responses. We note that this is nearly 1,000 more responses analyzed than those examined in Wiener et al. (2019).

To test whether these errors differed as a function of syllable token frequency and timing (and for the L2 group, test), the empirical log of the error ratio [log(probability error +0.5)/(acoustic error +0.5)] was calculated for each participant at each gate by syllable token frequency. More probability-based errors resulted in a positive log ratio whereas more acoustic-based errors resulted in a negative log ratio. Following Wiener and Ito (2016) and Wiener et al. (2019), gates 2 and 3 were treated as an “early” window (containing roughly half the errors) while the remaining gates were treated as a “late” window. This allowed for a roughly equal number of data points in the two windows. We note that this early/late grouping obscures important acoustic information and return to this limitation in our discussion.

**FIGURE 3:**
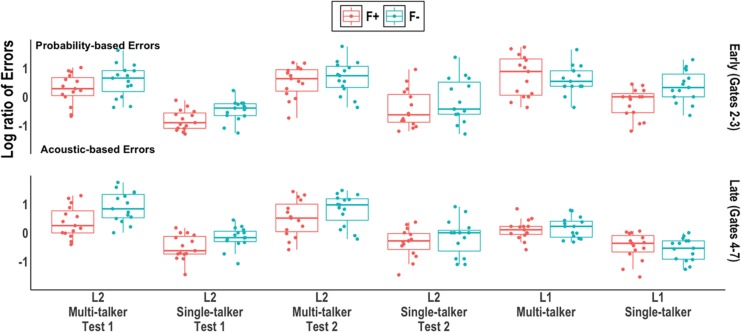
Log ratio of acoustic-based and probability-based errors by group and window. Individual participant means (points), group medians (solid line), and first and third quantiles (lower and upper hinges) are shown.

Separate models were run for the L1 and L2 groups; Frequency, Talker, and Window (and Test, for the L2 groups) were all contrast coded (+1, −1). Table 3 reports the model output, R code, and pairwise comparisons of interest. Figure 3 plots the individual log ratio by participant (dots), group distribution (box plots with median line) syllable frequency (color), and window.

**TABLE 3:** Mixed effect linear regression model on empirical log error ratio.

**L1 Group**	**Estimate**	**Std. error**	***t***	***p***
(Intercept)	0.08	0.04	1.86	0.065
Talker	0.31	0.04	6.92	<0.001
Window	0.28	0.04	6.26	<0.001
Talker:Window:Frequency	0.11	0.05	2.39	018
**Pairwise comparisons**				
Multi-talker – Single-talker	0.62	0.10	5.87	<0.001
Early (Gates 2-3) – Late (Gates 4-7)	0.56	0.09	5.70	<0.001
Early Multi (F−) – Early Single (F−)	0.24	0.18	1.31	0.893
Early Multi (F+) – Early Single (F+)	0.93	0.18	5.17	<0.001
Late Multi (F−) – Late Single (F-)	0.74	0.18	4.13	0.002
Late Multi (F+) – Late Single (F+)	0.57	0.18	3.21	0.036
**L2 Group**				
(Intercept)	0.10	0.04	2.49	0.015
Talker	0.48	0.04	−4.59	<0.001
Frequency	−0.14	0.03	11.35	<0.001
**Pairwise comparisons**				
Multi-talker – Single-talker	0.96	0.08	11.24	<0.001
(F−) – (F+)	0.28	0.06	4.28	<0.001

*L1 model: lmer[log ratio ∼ talker + window + talker:window:frequency + (talker| subject)]. L2 model: lmer[log ratio ∼ talker + frequency + (window| subject)].*

In the L1 model, main effects of Window and Talker were found; more probability-based errors were made in early gates compared to late gates (thus replicating Wiener and Ito, 2016; Wiener et al., 2019) and when listening to multi-talker speech compared to single-talker speech. A three-way interaction was also found between Window, Frequency, and Talker: the single-talker group primarily made probability-based errors on (F−) targets in the early window whereas the multi-talker group made probability-based errors on both (F+) and (F−) targets in the early and late windows.

In the L2 model, main effects of Frequency and Talker were found; more probability-based errors were made when listening to multi-talker speech compared to single-talker speech and on (F−) targets compared to (F+) targets. The syllable token frequency effect was found to be an aggregate effect across both talker groups and across both windows.

To summarize, the correct-syllable-incorrect-tone analyses revealed that both L1 and L2 listeners relied greater on probability-based tone processing when listening to multi-talker speech than when listening to single-talker speech. Two differences, however, were observed between the L1 and L2 groups. First, the L1 listeners primarily made probability-based errors in early gates, when there was less acoustic input compared to late gates. In contrast, L2 listeners made probability-based errors in both early and late gates. Additionally, when listening to multi-talker speech, L1 listeners made probability-based errors on both high token frequency (F+) and low token frequency (F−) syllables; when listening to single-talker speech, L1 listeners made probability-based errors primarily on (F−) targets. L2 listeners made more probability-based errors on (F−) syllables than (F+) syllables overall, resembling the L1 listeners in Wiener et al. (2019). However, unlike the L1 listeners in the current study, the probability-based errors did not vary across the talker conditions or times of the tests.

## Discussion

In this study, we set out to explore the flexibility often observed in bilingual spoken word recognition. Specifically, we examined how the perceptual trade-off between acoustic-based and knowledge-based processing of spoken words takes place under adverse listening conditions involving multi-talker, high variability speech. We used the gating paradigm to test the recognition of Mandarin words by L1 and L2 listeners. To explore how multi-talker speech affects the two modes of processing, we compared our results to those obtained from our previous study involving single-talker, low variability speech (Wiener et al., 2019). Additionally, to explore L2 listeners’ development of the two modes of processing, we tested the L2 participants before and after roughly 2 months of intermediate/advanced structured classroom learning.

We first found that in terms of syllable-tone word accuracy across gates, multi-talker speech, on average, adversely affected all listeners. Although both groups of listeners showed a small decrease in mean correct word recognition at gate 8 (i.e., the full acoustic signal) and across gates 2-7 (i.e., the truncated signal), this difference between multi-talker and single-talker conditions was statistically significant only for the L1 listeners. There are at least three reasons for the null effect of talker variability on the L2 listeners. One, we observed a floor effect. The L2 listeners tested on a single talker in Wiener et al. (2019) were already performing at a low level. Any decrease in performance due to the multiple talkers would therefore be negligible. This account is in line with previous experimental L2 tone research: even after multiple semesters of structured classroom learning, L2 listeners still struggle with tone perception and spoken word recognition (e.g., Wang et al., 1999; Hao, 2012, 2018; Wiener, 2017).

Two, we used a more complex experimental design. Lee et al. (2009, 2010b, 2013) only required listeners to identify one of four tones (i.e., a closed set with limited options). In the present study, however, we required listeners to spell out words and specify tones (i.e., a larger, open set and thus greater cognitive demand). Additionally, previous research by Lee and colleagues comparing L1 to L2 multi-talker tone perception treated “multiple” talkers as four (2009), six (2010a), and six (2013). The variability in four or six speakers is substantially smaller than the variability in 16 speakers as tested in the current study. We also note that our design compared single-talker to multi-talker speech across all gates, rather than within a single gate. Different results may be obtained by presenting the same multi-talker speech stimuli in two conditions: one with the talker fixed from trial to trial within each gate (fixed-talker condition) and the other with the talker varying from trial to trial within each gate (mixed-talker condition; e.g., Wong and Diehl, 2003).

Three, our statistical models accounted for the variance in L2 performance. The L2 listeners tested demonstrated a large amount of variability in their responses (Figures 1, 3). We assume our mixed effects models effectively captured this relatively large variance across participants by treating participants and items as random variables (see Baayen et al., 2008; Quené and Van den Bergh, 2008 for discussions). We note that less conservative statistical analyses that collapse across participants and items (e.g., *t*-tests and ANOVA with an alpha level of.05) revealed significant differences between the multi-talker and single-talker groups.

We next replicated our previous work (Wiener and Ito, 2016; Wiener et al., 2019) by demonstrating that from gate 2 (onset + 40 ms of the vowel) until gate 5 (onset + 160 ms of the vowel) L1 and L2 listeners recognized targets consisting of high token frequency (F+) syllables more accurately than those consisting of low token frequency (F−) syllables. Token frequency information therefore aided word recognition under adverse listening conditions for both L1 and L2 listeners. This finding is in line with previous research (e.g., Grosjean, 1980; Tyler, 1984), though the nature of our stimuli do not allow us to tease apart whether this effect is driven solely by token frequency, homophone density, or their combination (cf. Bradlow and Pisoni, 1999; Imai et al., 2005). As we noted in Wiener et al. (2019), answering that question would require testing infrequent syllables and uncommon words which the average L2 listener would not know or would require testing highly advanced learners with multiple years of L2 immersion (e.g., Pelzl et al., 2019).

Interestingly, during the first five gates containing the onset and up to 160 ms of the vowel, the L1 multi-talker group performed similarly to all the L2 groups while the L1 single-talker group was more accurate than all other groups. This difference between L1 groups was found to be primarily driven by the disproportionately reduced tone accuracy on high token frequency (F+) targets. These errors were also a large reason for the greater number of correct-syllable-incorrect tone errors for the multi-talker listeners as compared to the single-talker listeners. Thus, whereas the L2 listeners drew on learned syllable token frequency information at a relatively constant rate irrespective of the F0 variability in the stimuli (i.e., no interaction was observed), the L1 listeners demonstrated an interaction between frequency information and talker variability.

By gates 6-7, the L1 multi-talker and single-talker groups were more accurate than all the L2 groups while the L1 single-talker group was still more accurate than the L1 multi-talker group. The L1 listeners’ previously reported advantage of early word identification (Wiener and Ito, 2016; Wiener et al., 2019) was therefore neutralized by multi-talker speech until a sufficient amount of the acoustic signal − onset + 200 ms of the vowel − was available to listeners. In contrast, even with the majority of the acoustic input being present in gates 6 and 7, the L2 listeners did not show a difference between single-talker and multi-talker speech, or high- and low-token frequency items, or between test 1 and test 2. The lack of a talker effect for L2 listeners can again be partially attributed to listeners’ poor performance with a single talker, i.e., a floor effect. Given the relatively low accuracy (Figures 1, 2) in the single-talker conditions, it is possible that the added challenge of multi-talker speech could not further disrupt L2 performance when it was already substantially low.

Our analyses of correct-syllable-incorrect-tone response revealed similarities as well as important differences between the L1 and L2 listeners’ use of acoustic-based and probability-based processing. As predicted, overall both L1 and L2 listeners made more probability-based errors when listening to multi-talker speech than when listening to single-talker speech, suggesting that the added challenge of talker variability prompted both L1 and L2 listeners to rely to a greater extent on probability-based tone processing. Naturally, the L1 listeners were able to rely on more linguistic knowledge than the L2 listeners, which in turn contributed to more syllable-correct-tone-incorrect errors.

For the L1 listeners, errors occurred primarily in early gates (2-3) when listening to both single-talker and multi-talker speech. However, whereas single-talker speech triggered more probability-based errors only in (F−) targets, multi-talker speech triggered more probability-based errors across both (F+) and (F−) targets. That is, increased acoustic uncertainty due to multi-talker speech caused L1 listeners to rely on tonal probability information not only for low token frequency (F−) targets but also for high token frequency (F+) targets (cf. Wiener and Ito, 2016). In contrast, in late gates (4-7), probability-based errors decreased and acoustic-based error increased; with more acoustic input L1 listeners were able to use the acoustic signal to a greater extent despite the high variability introduced by multi-talker speech. That is, when more acoustic input became available, L1 listeners used their previous experience and internally stored pitch templates (Lee, 2017) to effectively engage in talker range normalization across a variety of different speakers.

For the L2 listeners, we had predicted two possibilities: First, without sufficient knowledge of tonal probabilities, L2 listeners would rely predominately on acoustic-based processing in early and late gates and only show more probability-based processing at test 2. Alternatively, L2 listeners would rely more on their knowledge of tonal distributions throughout the gates, however, limited this knowledge may be, because they are not as capable as L1 listeners in deciphering F0 information from multiple talkers. We found that L2 probability-based errors occurred in both early gates 2-3 and late gates 4-7, indicating that L2 listeners relied primarily on probability-based processing throughout the duration of a target. In other words, L2 listeners with relatively limited experience (and presumably less detailed internally stored pitch templates) were far less capable of resolving multi-talker F0 cues through talker normalization, even with almost-complete syllables in the later gates.

High talker variability, in addition to the fragmented input, therefore forced L2 listeners to rely on stored probability knowledge, which was presumably present in the participants tested in Wiener et al. (2019) but not fully utilized. Our results suggest that although L2 listeners can draw on learned syllable-tone probabilities like L1 listeners do, L2 listeners’ reliance on such distributional information for spoken word recognition is conditioned by the nature of the listening condition in a manner different from that of L1 listeners. With their limited experience with talker range normalization, L2 listeners are also less able to effectively transition from probability-based to acoustic-based processing as the duration of acoustic information increased. Thus, under adverse listening conditions such as multi-talker, high variability speech, listeners rely on other sources of linguistic information to resolve their perceptual uncertainty, such as distributional knowledge of speech sounds and their co-occurrences (e.g., Nixon et al., 2016; Wiener et al., 2018).

Although we had also predicted a difference between the two tests, our results − like those of the single-talker L2 listeners in Wiener et al. (2019)− did not show any effect involving tests. It is likely that our time window between the two tests was too short to see robust changes in learning. It is not uncommon for L1 English-L2 Mandarin learners to demonstrate an extended L2 tone learning plateau, which we may have captured in the present study (Wang et al., 1999; Hao, 2012, 2018; Wiener et al., 2020).

We acknowledge that our findings were likely affected to some degree by two features of our experimental design: individual differences and error coding. First, it is possible that individual differences in learning and perceptual abilities contributed to our results (e.g., Skehan, 1991; Perrachione et al., 2011; Bowles et al., 2016; Birdsong, 2018). Musical experience, working memory, motivation, among other individual traits were not controlled as rigorously as they should have been. Like most perceptual studies on L2 learners, we observed rather extensive variability across our participants (see Figures 1, 3). As we noted earlier, capturing this variability in statistical models (Barr et al., 2013) and in visualizations (Kampstra, 2008) is essential to better understanding the data. Related to our data, we also note that though participants responded to 48 items per gate, the number of participants tested per group was only 15, which may have underpowered our study and obscured certain effects (cf. Brysbaert and Stevens, 2018).

Second, as noted in the methods, our acoustic-based/probability-based error analysis approach was primarily designed for L1 listeners. Whereas the present study and Wiener et al. (2019) followed this approach in order to compare across studies, we acknowledge that the early/late window dichotomy was driven purely by a statistical concern to have roughly equal data points in the two windows. To what degree gate 4 is still early or late − and whether this differs between L1 and L2 listeners − is an open question that should be motivated by additional perception data. Furthermore, our classification of acoustic errors only considers the F0 onset and not the F0 offset. For example, an initial drop in F0 could indicate either Tone 4 or Tone 3 as both tones demonstrate a lower F0 offset as compared to the F0 onset. Our error coding approach was reasonable for L1 listeners who reached 50% tone accuracy by gate 2 in Wiener and Ito (2016) and by gate 3 in Wiener et al. (2019). In contrast, L2 listeners, did not reach 50% tone accuracy until gate 6 in both the present study and Wiener et al. (2019). Given L2 listeners’ poor tone perception, it seems likely that F0 offset may have contributed to the results.

In conclusion, our findings extend Wiener and Ito (2016) and Wiener et al. (2019) in demonstrating the additional challenge of multi-talker speech prompted L1 listeners to rely even greater on knowledge-based tone processing in acoustically adverse conditions for both high- and low-token frequency targets. However, once acoustic information became more available in later gates, L1 listeners were able to engage in effective talker normalization to identify tones from the acoustic input. L2 listeners likewise engaged in probability-based tone processing when the acoustic signal was highly variable in multi-talker speech. However, their response pattern differed from that of L1 listeners in that L2 listeners used probability-based processing in both early and late gates, presumably due to their relative ineffective use of talker normalization to process multi-talker tones.

## Data Availability Statement

The datasets generated for this study will not be made publicly available because the data came from participants enrolled in adult language classes at Ohio University and they did not consent to share their data. Summarized data will be made available by request.

## Ethics Statement

The studies involving human participants were reviewed and approved by the Ohio University IRB. The patients/participants provided their written informed consent to participate in this study.

## Author Contributions

SW created the experimental stimuli and design, conducted data analyses, and drafted the manuscript. C-YL assisted in stimuli creation, participant recruitment, data collection, and manuscript writing.

## Conflict of Interest

The authors declare that the research was conducted in the absence of any commercial or financial relationships that could be construed as a potential conflict of interest.
