# Supplementary material for: Multi-Talker Speech Promotes Greater Knowledge-Based Spoken Mandarin Word Recognition in First and Second Language Listeners
Source: Front Psychol. 2020 Feb 20;11:214. doi: 10.3389/fpsyg.2020.00214 (PMC7052525; doi:10.3389/fpsyg.2020.00214)
Supplement: Supplementary file 1 [file Table_1.DOCX]

Supplementary Material

# Stimuli

Target Condition Syllable token frequency Syllable homophone density

bao2 F+P+ 5.04 19

da4 F+P+ 5.31 10

gong1 F+P+ 4.93 16

ji1 F+P+ 5.30 70

qi2 F+P+ 5.29 48

ren2 F+P+ 5.48 12

shou3 F+P+ 4.95 12

si1 F+P+ 4.96 21

wu2 F+P+ 4.96 35

xiao3 F+P+ 5.10 24

yan2 F+P+ 4.75 38

zhu4 F+P+ 4.89 31

bao4 F+P- 5.04 19

da2 F+P- 5.31 10

gong3 F+P- 4.93 16

ji3 F+P- 5.30 70

qi1 F+P- 5.29 48

ren4 F+P- 5.48 12

shou1 F+P- 4.95 12

si3 F+P- 4.96 21

wu4 F+P- 4.96 35

xiao4 F+P- 5.10 24

yan4 F+P- 4.75 38

zhu2 F+P- 4.89 31

bin1 F-P+ 3.18 10

chai1 F-P+ 3.24 4

feng1 F-P+ 4.31 15

gua4 F-P+ 3.73 8

hong2 F-P+ 3.89 10

kao3 F-P+ 4.30 8

leng3 F-P+ 3.92 4

mao4 F-P+ 4.23 14

niao3 F-P+ 3.68 5

pang2 F-P+ 3.78 4

tie3 F-P+ 3.57 4

zhou1 F-P+ 4.35 14

bin4 F-P- 3.18 10

chai2 F-P- 3.24 4

feng3 F-P- 4.31 15

gua3 F-P- 3.73 8

hong4 F-P- 3.89 10

kao1 F-P- 4.30 8

leng4 F-P- 3.92 4

mao3 F-P- 4.23 14

niao4 F-P- 3.68 5

pang1 F-P- 3.78 4

tie1 F-P- 3.57 4

zhou2 F-P- 4.35 14

Calculations based on SUBTLEX-CH values; Syllable token frequencies reported in mean common log; Syllable homophone density represents number of homophonous morphemes associated with the syllable (independent of tone).

# Talker Characteristics

Home city/province Gender Age Time in U.S. (months)

Nanjing F 22 3

Shandong F 22 3

Shenyang F 19 11

Hubei F 23 10

Beijing F 22 3

Liaoning F 30 47

Shandong F 28 22

Zhejiang F 25 6

Shanghai M 22 3

Nanjing M 21 50

Shanghai M 32 32

Heilongjiang M 22 8

Hubei M 28 16

Shaanxi M 33 28

Anhui M 23 12

Chongqing M 26 33

# R model comparisons

Analysis 1 (Table 1) correct syllable-tone at gate 8

Model 1: glmer(corect ~ group+(1|subject)+(1|token), family="binomial")

Model 2: glmer(corect ~ group+(1|subject)+(talker|token), family="binomial") *[Final model]*

Model 1—Model 2: ΔAIC = -35.3, χ^2^(2) = 52.32, *p* < .001

Analysis 2 (Table 2) correct syllable-tone at gates 2-3

Model 1: glmer(corect ~ group+frequency+(1|subject)+(1|token), family="binomial")

Model 2: glmer(corect ~ group*frequency+(1|subject)+(1token), family="binomial")

Model 1—Model 2: ΔAIC = -1.4, χ^2^(5) = 10.49, *p* = .062

Model 3: glmer(corect ~ group+frequency+probability+(1|subject)+(1|token), family="binomial")

Model 1—Model 3: ΔAIC = -2.0, χ^2^(1) = 2.08, *p* = .149

Model 4: glmer(corect ~ group+frequency+(1|subject)+(talker|token), family="binomial")

Model 1—Model 4: ΔAIC = -87.0, χ^2^(2) = 91.08, *p* < .001

Model 5: glmer(corect ~ group+frequency+(frequency|subject)+(talker|token), family="binomial") *[Final model]*

Model 4—Model 5: ΔAIC = -3.4, χ^2^(2) = 7.38, *p* = .025

correct syllable-tone at gates 4-5

Model 1: glmer(corect ~ group+frequency+(1|subject)+(1|token), family="binomial")

Model 2: glmer(corect ~ group*frequency+(1|subject)+(1token), family="binomial")

Model 1—Model 2: ΔAIC = -2.6, χ^2^(5) = 9.60, *p* = .087

Model 3: glmer(corect ~ group+frequency+probability+(1|subject)+(1|token), family="binomial")

Model 1—Model 3: ΔAIC = -0.3, χ^2^(1) = 1.67, *p* = .195

Model 4: glmer(corect ~ group+frequency+(1|subject)+(talker|token), family="binomial")

Model 1—Model 4: ΔAIC = -501.0, χ^2^(2) = 504.98, *p* < .001

Model 5: glmer(corect ~ group+frequency+(frequency|subject)+(talker|token), family="binomial") *[Final model]*

Model 4—Model 5: ΔAIC = 2.2, χ^2^(2) = 2.14, *p* = .343

correct syllable-tone at gates 6-7

Model 1: glmer(corect ~ group+frequency+(1|subject)+(1|token), family="binomial")

Model 2: glmer(corect ~ group*frequency+(1|subject)+(1token), family="binomial")

Model 1—Model 2: ΔAIC = -1.0, χ^2^(5) = 9.28, *p* = .098

Model 3: glmer(corect ~ group+frequency+probability+(1|subject)+(1|token), family="binomial")

Model 1—Model 3: ΔAIC = -1.1, χ^2^(1) = 3.06, *p* = .080

Model 4: glmer(corect ~ group+frequency+(1|subject)+(talker|token), family="binomial")

Model 1—Model 4: ΔAIC = -536.0, χ^2^(2) = 504.64, *p* < .001

Model 5: glmer(corect ~ group+frequency+(frequency|subject)+(talker|token), family="binomial") *[Final model]*

Model 4—Model 5: ΔAIC = -14.0, χ^2^(2) = 17.74, *p* < .001

Analysis 3 (Table 3) correct syllable-incorrect tone empirical log error ratio

L1 Model

Model 1: lmer(log ratio ~ talker+(1|subject))

Model 2: lmer(log ratio ~ talker+window+(1|subject))

Model 1—Model 2: ΔAIC = -31.13, χ^2^(1) = 33.13, *p* < .001

Model 3: lmer(log ratio ~ talker*window+(1|subject))

Model 2—Model 3: ΔAIC = 2, χ^2^(1) = 0.18, *p* = .670

Model 4: lmer(log ratio ~ talker+window+frequency+(1|subject))

Model 2—Model 4: ΔAIC = -0.58, χ^2^(1) = 1.38, *p* = .239

Model 5: lmer(log ratio ~ talker+window+talker:window+(1|subject))

Model 2—Model 5: ΔAIC = -1.82, χ^2^(1) = 1.23, *p* = .267 .

Model 6: lmer(log ratio ~ talker+window+talker:window:frequency+(1|subject))

Model 2—Model 6: ΔAIC = -26.10, χ^2^(1) = 28.10, *p* < .001

Model 7: lmer(log ratio ~ talker+window+talker:window:frequency+(talker|subject)) *[Final model – additional random effects would not converge]*

Model 6—Model 7: ΔAIC = 3.87, χ^2^(2) = 0.130, *p* = . 937

L2 model

Model 1: lmer(log ratio ~ talker+(1|subject))

Model 2: lmer(log ratio ~ talker+frequency+(1|subject))

Model 1—Model 2: ΔAIC = -5.50, χ^2^(1) = 7.50, *p* = .006

Model 3: lmer(log ratio ~ talker*frequency +(1|subject))

Model 2—Model 3: ΔAIC = 2.01, χ^2^(1) = 0.07, *p* = .932

Model 4: lmer(log ratio ~ talker+frequency+window+(1|subject))

Model 2—Model 4: ΔAIC = -3.16, χ^2^(1) = 3.78, *p* = .052

Model 5: lmer(log ratio ~ talker+frequency+talker:frequency+(1|subject))

Model 2—Model 5: ΔAIC = 2.02, χ^2^(1) = 0.02, *p* = .888

Model 6: lmer(log ratio ~ talker+frequency+talker:frequency:window+(1|subject))

Model 2—Model 6: ΔAIC = 1.77, χ^2^(1) = 0.231, *p* = .630

Model 7: lmer(log ratio ~ talker+frequency+(window|subject)) *[Final model – additional random effects would not converge]*

Model 2—Model 7: ΔAIC = -2.13, χ^2^(2) = 6.127, *p* = .046
